# Does long-term recreational gymnastics prevent injurious falls in older women? A prospective 20-year follow-up

**DOI:** 10.1186/s12877-020-1428-0

**Published:** 2020-02-01

**Authors:** Kirsti Uusi-Rasi, Saija Karinkanta, Pekka Kannus, Kari Tokola, Harri Sievänen

**Affiliations:** 1 UKK Institute for Health Promotion Research, Kaupinpuistonkatu 1, FI-33500 Tampere, Finland; 2 Medical School, University of Tampere, Tampere, Finland; 3 Department of Orthopedics and Trauma Surgery, Tampere University Hospital, Tampere, Finland

**Keywords:** Exercise, Physical activity, Falls, Fractures, Older women

## Abstract

**Background:**

Exercise interventions focusing on balance and strength training have been shown to be effective for falls prevention. The aim of this 20-year register-based follow-up was to examine whether long-term participation in recreational female gymnastics is associated with a lower risk of medically-attended injurious falls.

**Methods:**

Health care register data of 187 women (103 recreational gymnasts and 84 sedentary controls) from the original cohort of 243 women were assessed. The mean age (sd) at baseline was 62.8 (5.4) years and the mean follow-up time was 19.4 (2.7) years (range from 5.6 to 21.0 years).

Injurious falls were scrutinized from medical records. An injurious fall was defined as an event in which falling was mentioned as a reason for making contact with health-care professionals. Negative binomial regression was used to estimate incidence rate ratios (IRR) for injurious falls, and Cox-regression models for calculating hazard ratios (HR) for injured fallers with the control group as reference.

**Results:**

Recreational gymnasts had about 30% less injurious falls compared to controls, the mean IRR (95% CI) being 0.71 (0.51 to 0.96). The HR for injured fallers was 0.73 (0.52 to 1.02) favoring the recreational gymnasts. There were no statistically significant between-group differences for fractures.

**Conclusions:**

Long-term recreational gymnastics appears to reduce the risk of injurious falls in old age.

## Background

Falls in older adults are a major economic and public health concern, and ageing of the population further escalates the expenses involved. Falls and fear of falling can reduce physical activity and functional capacity, impair the ability to perform daily activities, reduce quality of life, and increase the risk of social isolation and institutionalization [1, 2].

Focusing on strategies reducing the risk of falls and related injuries in the older population would not only decrease pain and disability of individual older adults but also reduce the costs to society significantly [3]. Even if the incidence of falls and fall-related injuries were to remain stable, absolute numbers of such injuries would continue to rise because the number of people living into older age is increasing; centenarians are no longer unusual [4].

History of falls is a strong indicator for increased risk of fractures [5], and thus preventing falls is crucial in the prevention of injuries and fractures in the older population [6]. Epidemiologic evidence suggests that physical activity is beneficial in the reduction of fragility fractures [7–9], and exercise training has been found to be the single most effective method of preventing falls and related injuries in community-dwelling older people [10–13]. Therefore, it is important to raise awareness among older adults about individual fall risk and motivate them to exercise regularly and remain physically active. Balance and functional training, as well as multicomponent exercise comprising of balance and strength training have been shown to be most effective preventive measures [11–13]. Furthermore, both group and home-based training interventions have been successful [14–17]. However, less is known about long-term effects of exercise training. Karinkanta et al. showed that the incidence of injurious falls was lower in the group focusing on resistance and balance-jumping training 5 years after the intervention, while groups with solely resistance or balance training did not differ from controls [18].

We have previously shown that in postmenopausal women, regular long-term participation in recreational gymnastics was associated with better overall physical fitness through improved muscle performance, agility and balance. Recreational gymnastics was also positively associated with greater bone mass and bone strength especially in the weight-bearing lower limbs [19, 20]. Briefly, recreational female gymnastics is light to moderate in intensity and emphasizes a springy gait and body flexibility. It also includes more strenuous exercise causing elevation of heart rate and including muscular strength training, but it does not have any high impact training as is common in standard aerobics or apparatus gymnastics. Finnish folk dancing is, in turn, aerobic dancing with brisk turns and light jumps that create low to moderate impact. Women who participated in folk dancing were also active in recreational gymnastics. The above described training is referred as recreational gymnastics in this report. Recreational gymnastics is a combination of balance, agility and strength training, although not to the same extent or intensity as in gym training or Tai Chi. Further, recreational gymnastics is not specifically planned for falls’ or fracture prevention but rather for improving general wellness.

In this 20-year follow-up study, we used the same female cohort to assess whether older women who had been engaged in recreational gymnastics for decades differed from their sedentary counterparts in terms of medically-attended fall-related injuries. A lower incidence of fall-related injuries would indicate that recreational gymnastics has potential and thus add to the existing body of evidence that certain types of exercises are particularly beneficial in preventing injurious falls.

## Methods

### Participants

At baseline, all participants were healthy, postmenopausal, non-smoking women aged from 55 to 83 years. Recreational gymnasts and folk dancers were recruited from local clubs while their sedentary controls were obtained via a local newspaper advertisement. Exercising women had trained at least 20 years in recreational gymnastics or folk dance. Any woman who had competed in apparatus gymnastics at any age was excluded from the original study [19]. Women who participated no more than once a week in light or moderate exercise, that was not gymnastics or folk dancing were eligible as sedentary controls [19].

The participants’ history in recreational gymnastics was determined for 10-year periods from the age of 16 to 45 years, and for 5-year periods thereafter (average duration of one session, number of sessions a week, training months a year, and training years in each period). The controls answered the same questions in order to verify that they fulfilled the selection criteria of the study.

The study protocol was approved by the Human Ethics Committee of the Tampere Region, University of Tampere, Finland (approval 53/2017). The use of the patient-register data was further approved by the Department of Social Services and Health Care of the City of Tampere. Be it also noted that this 20-year follow-up focused solely on medical records without any personal contact with the former participants.

### Procedure

**Fig. 1:**
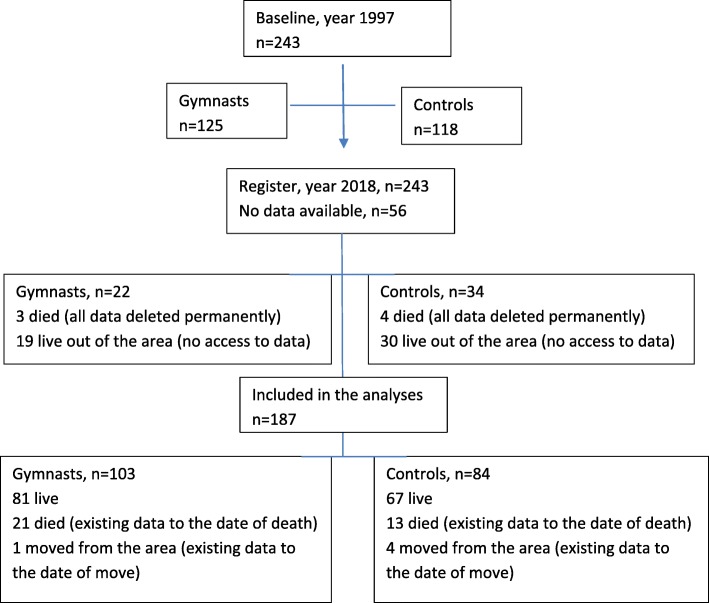
Flow chart of the register-data collection

This 20-year follow-up was conducted for all women who were still living in Tampere and had participated in the original study in 1997 [19]. From the original cohort of 243 women, 187 (77%, 103 recreational gymnasts and 84 sedentary controls) were included in this register-based follow-up study. Data was not available for 56 women (Fig. 1). The Pegasos patient medical records of the City of Tampere (Pegasos Patient Information System, CGI, Finland) were scrutinized for fall-related health services utilization including hospitalizations for all participants during the follow-up period starting from September 1997 to April 2018; the years between 1997 and 2002 were manually examined from paper files, while more recent data was available in digital format**.** A single researcher (KU-R) did all the data extraction from health care registers.

### Outcomes

The primary outcome of the present study was the rate of medically-attended injurious falls including fractures during the 20-year follow-up period. In addition, fall-induced fractures and the rate of injured fallers and fallers with fractures were evaluated as secondary outcomes.

Medically attended fall injuries were defined according to medical care and conformed to a recommended definition of a fall [21], and included injuries that have required medical/health professional examination, or emergency/inpatient treatment, regardless of the severity of injury [22].

All contacts with the local Finnish health care system mentioning “fall”, “fall-related”, or “fall-injury” as reason for the contact were recorded. Injurious falls included injuries, such as bruises, abrasions, contusions, sprains, fractures, and head injuries. An injured faller was defined as a person who had contacted the health care system at least once due to a fall during the 20-year follow-up period. Information concerning the original study group assignment was added to the data only after all injurious falls were recorded.

### Statistical analysis

Follow-up time for injurious falls and fallers was calculated from the baseline (September 1997) to the end of the follow-up period (April 2018).

Injurious fall incidence rates were calculated as the total number of falls divided by the time over which injurious falls were monitored in both groups. Negative binomial regression was used to estimate incidence rate ratios (IRR) for injurious falls and falls with fracture, and Cox-regression models for calculating hazard ratios (HR) for injured fallers and fallers with fractures, using the control group as reference. All analyses were adjusted for baseline age, height and weight as possible confounding factors. All non-significant confounders were removed one by one from the final models if their *p*-values exceeded 0.20. Negative binomial regression models were also adjusted for follow-up time.

SPSS 25 statistical software was used for all statistical analyses. *P*-values were 2-sided and those less than 0.05 were considered statistically significant.

## Results

The baseline group descriptions are given in Table 1. The recreational gymnasts were slightly older and lighter than the controls. The follow-up comprised data for 187 of the original 243 women (77%). The mean (sd) follow-up time was 19.4 (2.8) years ranging from 5.6 to 21.0 years, totalling to 3635 person-years. Clinical characteristics of 56 women with no follow-up data did not differ statistically significantly from the 187 included women.

**Table 1:** Group characteristics, mean (SD)

	Sedentary controls *n* = 84	Recreational gymnasts *n* = 103	All *n* = 187	Missing cases *n* = 56
Age at baseline 1997, years	62.1 (4.6)	63.3 (5.9)*	62.8 (5.4)	62.3 (5.2)
Height at baseline, cm	161.4 (5.8)	160.8 (5.2)	161.1 (5.5)	162.2 (5.3)
Weight at baseline, kg	68.4 (10.4)	66.7 (9.6)*	67.5 (10.0)	67.6 (9.9)
BMI	26.3 (3.8)	25.8 (3.5)	26.0 (3.6)	25.7 (3.3)
Age at register 2018, years	81.3 (5.1)	82.8 (5.6)	82.1 (5.4)	
Follow-up time, years	19.3 (3.1)	19.6 (2.4)	19.4 (2.7)	
Range of follow-up, years	5.6–20.7	9.3–21.0	5.6 – 21.0	

*Statistically significant difference between groups, *p* < 0.05

### Injurious falls

During the 19.4 (2.8) years follow-up, 378 injurious falls were recorded among 135 (72.2%) women. Only less than 30% of women did not sustain any injurious fall. One fourth sustained a single injurious fall and nearly a half of women had at least two injurious falls. About a third of the women sustained a fracture as a consequence of falling (Table 2).

**Table 2:** Injurious falls and injured fallers between 1997 and 2018, rate of injurious falls, incidence rate ratio (IRR) and hazard ratio (HR) adjusted for age, height and weight at baseline (95% CI)

	Sedentary controls *n* = 84	Recreational gymnasts *n* = 103	All *n* = 187
All injurious falls, n	194	184	378
All injurious falls/10 years	1.20	0.91	
All falls with fractures	39	63	
All falls with fractures/10 years	0.24	0.31	
Age at first injurious fall, years	72.0 (6.4)	72.7 (8.1)	72.3 (7.3)
Non-Faller, n (%)	17 (20.2)	35 (34.0)	52 (27.8)
Injured faller, n (%)	67 (79.8)	68 (66.0)	135 (72.2)
Faller (1 fall)	23 (27.3)	24 (23.3)	47 (25.1)
Faller (2 falls)	14 (16.7)	19 (18.4)	33 (17.6)
Faller (multiple falls)	30 (35.7)	25 (24.3)	55 (29.4)
Fallers with fractures, n (%)	29 (34.5)	38 (36.9)	67 (35.8)
Fallers with 1 fall resulting fracture	24 (28.6)	25 (24.3)	49 (26.2)
Fallers with 2 falls resulting fractures	2 (2.4)	7 (6.8)	9 (4.8)
Fallers with multiple falls resulting fractures	3 (3.6)	6 (5.8)	9 (4.8)
Age at first fracture, years	76.2 (6.8)	72.4 (7.5)	74.1 (7.4)
IRR (95% CI)			*P*-value
All injurious falls	1	0.71 (0.52 to 0.96)*	0.026
Falls with fractures	1	1.32 (0.81 to 2.17)	0.27
HR (95% CI)			
All injured fallers	1	0.73 (0.52 to 1.02)	0.068
Fallers with fractures	1	1.16 (0.72 to 1.89)	0.54

*Statistically significant difference between groups, *p* < 0.05

### Fractures

There were 67 fallers with 113 fractures. The most common fractures were upper limb fractures (33 in recreational gymnasts and 15 in controls), while the most severe hip fractures occurred equally in both groups (6 in each). Head injuries were also quite common, 61 head injuries including two facial fractures required medical attention. Severe fall-induced injuries are compiled in Table 3.

**Table 3:** Fractures and head injuries caused by falls, n (%)

	Sedentary controls *n* = 84	Recreational gymnasts *n* = 103	Total *n* = 187
Hip/pelvic area	6 (7.1)	6 (4.6)	12 (6.4)
Upper limb (wrist and humerus)	15 (17.9)	33 (32.0)	48 (25.7)
Lower limb (tibia, fibula, ankle)	2 (2.4)	7 (6.8)	9 (4.8)
Spine	5 (5.9)	5 (4.9)	10 (5.3)
Clavicle and rib	3 (3.6)	2 (1.9)	5 (4.9)
Metacarpals and Phalanges	3 (3.6)	6 (5.8)	9 (4.8)
Metatarsals and toe bones	1 (1.2)	0	1 (0.5)
Facial bones	1 (1.2)	1 (1.0)	2 (1.1)
Head injuries (including swellings and bruises)	28 (33.3)	31 (30.1)	59 (31.6)

### Comparison between recreational gymnasts and controls

**Fig. 2:**
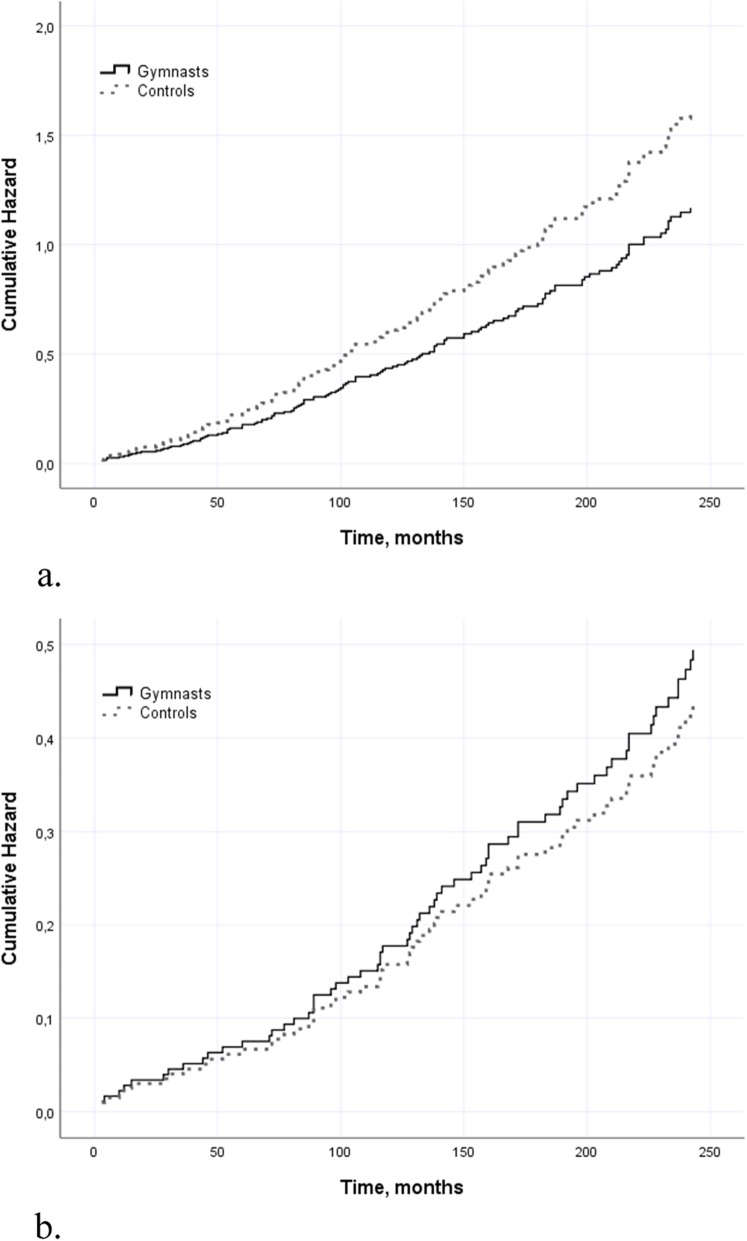
Hazard ratio for injured fallers with injuries including fractures (panel **a**) and for fallers with fractures only (panel **b**) among recreational gymnasts and sedentary controls

Recreational gymnasts had nearly 30% less injurious falls than controls; incidence rate ratio (IRR; 95% CI) 0.71 (0.52 to 0.96). However, the between-group difference in fall-induced fractures was not statistically significant (Table 2). The Cox model showed a 27% lower rate of injured fallers in recreational gymnasts than in controls (HR 0.73; 0.52 to 1.02), but the risk was the opposite in fallers with fractures. However, these differences were not statistically significant (Table 2, Fig. 2). Recreational gymnasts were on average 4 years younger than sedentary controls when they sustained their first fracture.

As a sensitivity analysis, the above analyses were repeated in age-based tertiles and the results were in line with the results observed in the entire group (data not shown).

## Discussion

There was a statistically significant difference in medically-attended injurious falls between the recreational gymnasts and controls, the former having about 30% less injurious falls during the 20 years’ follow-up. However, the 27% between-group difference in injured fallers did not reach statistical significance. Regarding the fractures and fallers with a fracture, there was no significant group difference either.

Previously, clinical trials have shown that resistance and balance training reduces the risk of falls, especially that of injurious falls [10, 11]. In their recent Cochrane-review, Sherrington et al. reported that exercise reduced the rate of falls by 23% on average. Most effective exercise programs primarily comprised balance and functional exercises, while programs including multiple exercise categories (typically balance and functional exercises, as well as resistance exercises) may be beneficial as well [13]. However, individual trials that directly compared higher with lower doses of similar exercise were too few for definitive conclusions [13]. However, in general exercise programs of a higher dose seem to have larger effects. Tai Chi had a greater impact on the rate of falls when classes were delivered twice rather than once per week [23] and Kemmler et al. [24] found greater effects on the rate of falls using a more intensive program delivered twice a week compared with a low intensity program delivered once a week. In their previous review Sherrington et al. concluded that although the literature does not provide a clear cut-off there is an indication for greater benefits from higher doses of exercise, and they recommended that exercise should be undertaken for at least 2 h per week on an ongoing basis [25, 26].

Recreational gymnastics was executed at least twice a week. In most exercise trials the frequency was once or twice a week. Our results are in line with these findings. Although recreational gymnastics is rather light in general, it is a combination of strength, agility and balance training and executed at least twice a week. Most likely, this is sufficient to improve physical functioning.

Although the recreational gymnasts in our study had less injurious falls in general, they showed a trend for more upper limb fractures. While this may seem contradictory, this finding is not unique. Physical activity was associated with a reduced risk of hip fractures in the SOF-study, but the risk of wrist fractures was slightly increased among physically active older women [27]. In the Tromsø study, high rates of physical activity were related to a 50% increased relative risk of upper limb fractures [28]. Also, a long-term follow-up study of a Finnish cohort suggested more wrist fractures among physically active postmenopausal women compared to inactive women of similar age [29]. Frequent walking has been associated with increased risk of fractures among older adults [30], but active commuting among middle-aged women was associated with a lower wrist fracture risk [31]. A recent meta-analysis of RCTs examining exercise and fracture risk showed that exercise is generally related to reduced fracture risk [32].

As discussed previously [27, 28], some explanations for the increased risk of upper limb fractures in physically active older persons may be debated. Recreational gymnasts were more agile at baseline and 6 years later [19, 20], and this benefit may have been maintained in older age. Thus, when slipping or tripping, recreational gymnasts reacted fast enough to extend their arm to absorb the impact energy resulting in more wrist fractures in place of other injuries, such as hip fracture or head injury. Having a feeling of good performance may also predispose not only to higher exposure time to physical activity [31], but also to higher walking speed and more risky behavior. Moreover, when stumbling at faster gait speed the impact force on falling is likely greater and possibly sufficient to fracture the upper limb bones. In this study, recreational gymnasts were about 4 years younger than sedentary controls when sustaining the fall-induced fracture, which may support the notion of faster walking speed while falling. However, instead of avoiding moving outside or declining physical activity, older people should pay attention to safe walking e.g. by using practical footwear, walking poles and/or shoe grip spikes. It is equally important to identify fall risks at home and mitigate them.

The strengths of this study are the long follow-up time and comprehensive evaluation of participants’ specific history in recreational gymnastics (determined in 10-year periods from the age of 16 to 45 years, and 5-year periods thereafter including duration and number of sessions per week, training months and years). The eligibility of controls as being sedentary was verified by their answers to the same questions. In addition, injurious falls were verified from medical records, which increased the reliability of the data. The types of injuries and the treatment provided were well described in the records, including the most severe traumas with exact ICD-codes, but the location (e.g. outdoors/indoors) or circumstances of the fall were not always mentioned.

One limitation of the study is that while the recreational gymnasts had exercised an average of 33 years at baseline in 1997, we had no information about their physical activity, participation in gymnastics, physical performance or mobility status after the 6-year follow-up in 2003. It is possible that participants’ physical activity and performance have declined with aging, chronic diseases, other incident health problems have increased or social issues changed, but we have no measured data to support this phenomenon. Physical activity was not recorded systematically in the medical records, nor was physical performance or functioning. However, we decided not to invite the former participants to the 20-year follow-up measurements because it was expected that only the women in good physical condition would have been able to participate, and this would have biased the analyses.

Another limitation is that medical records were available only for the women living in City of Tampere, and who had contacted the public health care system due to injuries. It is possible that some women may have sought treatment from private health care services. However, in Finland, senior citizens after retirement no longer remain under the domain of occupational health care, but fall within the public health care service system. Practically all 52 women with no mention of an injurious fall in their medical records had contacted the public health care system for some other health reason. Some injurious falls were treated elsewhere when travelling, but the aftercare was carried out in the local health care center or hospital, and these cases could be counted. Also, because we did not have access to medical records of surrounding municipalities, we had to exclude the women living in these neighborhoods. In addition, records of 7 deceased women had already been permanently deleted including the cause of death. However, this missing information was not likely to alter the findings because women with and without available register data were similar in their baseline characteristics, and the proportion of missing data (23%) was relatively small.

We did not have information about falls which did not require contact with the health care system. Most likely, these occurrences were falls with no consequences, or they resulted in mild injuries causing no long-term harm, disability or pain, and therefore the person did not consider it necessary to visit a health center to see a physician. We had permission only to access fall-related injurious data, not to the other health data, e.g. other diseases or medication during the follow-up. Although all participants were relatively healthy at baseline, the possibility that within-group changes in health status were not similar cannot be ruled out. It is well known that higher amounts of physical activity and fitness are associated with better health status in general. Furthermore, at baseline about half of the participants used estrogen replacement therapy, but there was no difference in injurious fallers between previous estrogen users and non-users. Estrogen was not associated with physical functioning at baseline either, but was associated with greater bone mass [19, 20]. However, users of estrogen replacement therapy were equally divided into both groups.

Although the health benefits of regular physical activity are well established, poor exercise adherence and compliance with physical activity recommendations is very common in the elderly population [33]. On the other hand, long-term physical activity at a younger age predicts physical activity in old age [34]. Also, in intervention trials, adherence and compliance remain fairly good as long as the training is supervised, but after the intervention, the adherence tends to return to the baseline. The benefit of recreational gymnastics is that it is offered by several clubs in Finland with low semester costs and exercise sessions are held in residential areas, making the training easily accessible, feasible and safe to perform. Despite being light to moderate by intensity, long-term regular participation may compensate for the benefits of more intensive training, which often is discontinued after a short period. Further research is needed to understand and establish the impact of different exercise programs and fall prevention interventions on fall-related fractures and injuries. Also, novel methods are needed to support older adults to continue physical activity after supervised interventions and promote life-long exercise training habits for maintaining physical functioning, balance and agility in older age.

## Conclusions

Long-term light-to-moderate intensity recreational gymnastics comprising muscle strength, balance and mobility training seems to reduce the risk for medically-attended injurious falls in older age.

## Data Availability

The datasets used and/or analyzed during the current study are available from the corresponding author on reasonable request.

## Abbreviations

CI: Confidence interval
HR: Hazard ratio
IR: Incidence rate ration
